# The effect of perinatal anxiety on bronchiolitis is influenced by polymorphisms in ROS-related genes

**DOI:** 10.1186/1471-2466-14-154

**Published:** 2014-09-29

**Authors:** Eun Lee, Hyoung Yoon Chang, Kyung-Sook Lee, Dong In Suh, Ho-Sung Yu, Mi-Jin Kang, In Ae Choi, Jinah Park, Kyung Won Kim, Youn Ho Shin, Kang Mo Ahn, Ja-Young Kwon, Suk-Joo Choi, Kyung-Ju Lee, Hye-Sung Won, Song I Yang, Young-Ho Jung, Hyung Young Kim, Ju-Hee Seo, Ji-Won Kwon, Byoung-Ju Kim, Hyo-Bin Kim, So-Yeon Lee, Eun-Jin Kim, Joo-Shil Lee, Katherine M Keyes, Yee-Jin Shin, Soo-Jong Hong, the COCOA study group

**Affiliations:** Department of Pediatrics, Childhood Asthma Atopy Center, Research Center for Standardization of Allergic Diseases, Asan Medical Center, University of Ulsan College of Medicine, 88 Olympic-ro 43-gil, Songpa-gu, Seoul 138-736 Korea; Department of Psychiatry, Ajou University College of Medicine, Gyeonggi-do, Korea; Department of Rehabilitation, Hanshin University, Gyeonggi-do, Korea; Department of Pediatrics, Seoul National University College of Medicine, Seoul, Korea; Asan Institute for Life Sciences, University of Ulsan College of Medicine, Seoul, Korea; Sewon Infant Child Development Center, Seoul, Korea; Department of Pediatrics, Severance Children’s Hospital, College of Medicine, Yonsei University, Seoul, Korea; Department of Pediatrics, CHA Medical Center, CHA University School of Medicine, Seoul, Korea; Department of Pediatrics, Samsung Medical Center, Sungkyunkwan University School of Medicine, Seoul, Korea; Department of Obstetrics and Gynecology, Yonsei University College of Medicine, Seoul, Korea; Department of Obstetrics and Gynecology, Samsung Medical Center, Sungkyunkwan University School of Medicine, Seoul, Korea; Department of Obstetrics and Gynecology, Asan Medical Center, University of Ulsan College of Medicine, Seoul, Korea; Department of Pediatrics, Pusan National University Yangsan Hospital, Yangsan, Korea; Department of Pediatrics, Korea Cancer Center Hospital, Seoul, Korea; Department of Pediatrics, Seoul National University Bundang Hospital, Seongnam, Korea; Department of Pediatrics, Inje University Haeundae Paik Hospital, Busan, Korea; Department of Pediatrics, Inje University Sanggye Paik Hospital, Seoul, Korea; Department of Pediatrics, Hallym University Sacred Heart Hospital, Hallym University College of Medicine, Anyang, Korea; Allergy TF, Department of Immunology and Pathology, Korea National Institute of Health, Cheongwon, Korea; Department of Epidemiology, Columbia University Mailman School of Public Health, New York, NY USA; Department of Psychiatry, Gangnam Severance Hospital, Yonsei University College of Medicine, 250 Seongsandong, Seodaemun-gu, Seoul 120-752 Korea

**Keywords:** Anxiety, Bronchiolitis, CD14, Perinatal, Glutathione *S*-transferase, Polymorphism, Respiratory tract infection

## Abstract

**Background:**

Exposure to perinatal anxiety affects disease susceptibility in offspring but studies on the association between perinatal anxiety and gene polymorphisms are lacking. This study aimed to elucidate the interaction between perinatal anxiety and polymorphisms in antioxidant defense and innate immunity genes on the development of respiratory tract infections (RTIs) during early infancy.

**Methods:**

Trait anxiety levels in 440 women were assessed by the State-Trait Anxiety Inventory during late gestation. The occurrence of RTIs, including bronchiolitis, during the first year of life was assessed by parent-reported doctor diagnosis. Polymorphisms in glutathione S-transferase P-1 (*GSTP1*, rs1695) and *CD14* (rs2569190) were genotyped using the TaqMan assay. Copy number variations of *GSTT1* were measured by real-time polymerase chain reaction.

**Results:**

Exposure to high levels of perinatal anxiety increased the risk of bronchiolitis in the first year of life (adjusted odds ratio [aOR], 1.30; 95% confidence interval [CI]: 1.00–1.80), in particular among children with the AG + GG genotype of *GSTP1* or the *GSTT1* null genotype (aOR 3.36 and 2.79). In infants with the TC + CC genotype of *CD14*, high levels of perinatal anxiety were associated with an increased risk of upper RTI, lower RTI, and bronchiolitis (aOR 2.51, 4.60, and 4.31, respectively).

**Conclusions:**

Perinatal maternal anxiety levels affect the occurrence of bronchiolitis in offspring. The effect of perinatal anxiety on the occurrence of bronchiolitis during infancy was influenced by genetic polymorphisms in antioxidant defense and innate immunity genes.

**Electronic supplementary material:**

The online version of this article (doi:10.1186/1471-2466-14-154) contains supplementary material, which is available to authorized users.

## Background

Environmental factors during early life influence the development of the immune system and physiology, and change susceptibility to disease in later life [1]. Recent studies reveal that perinatal maternal stress and anxiety affect susceptibility to infectious diseases in later life [2]. Respiratory tract infections (RTIs) account for the majority of morbidity and mortality during infancy and thus pose a worldwide burden that can be prevented by the modification of environmental factors [3, 4].

The mechanisms underlying the associations between exposure to perinatal maternal anxiety and an increased risk of RTIs are not fully elucidated. Perinatal maternal anxiety increases glucocorticoids in pregnant women, which can cross the placental barrier. This may affect fetal programming through immunomodulation [5], and perinatal anxiety may cause epigenetic changes in the offspring leading to an increased vulnerability to neurodevelopmental diseases in later life [6]. However, no study has directly demonstrated the possible mechanisms underlying the association between exposure to perinatal maternal anxiety and development of RTIs in offspring.

Perinatal anxiety and RTIs share a common pathway of oxidative stress [7, 8]. Exposure to perinatal anxiety increases the serum levels of glucocorticoids in offspring [9], generating higher levels of reactive oxygen species (ROS). During RTIs, high levels of ROS are generated in the epithelium of the respiratory tract. This increased oxidative stress is associated with alterations in the immune response and affects fetal programming [10, 11].

The glutathione S-transferase (GST) subfamily plays an important role in the protection against oxidative stress by catalyzing the conjugation of many compounds with reduced glutathione [12]. Polymorphisms in the GST genes, *GSTP1* (rs1695) and *GSTT1*, affect the ability to deal with excessive oxidative stress due to the resultant altered activity of the GST enzymes [12]. In subjects with the AG + GG genotype of *GSTP1* (rs1695), the enzymatic activity of GSTP1 is partially reduced, compared to subjects with the AA genotype [12]. The null genotype of *GSTT1* is associated with an absence of enzyme activity [12]. CD14 is essential for host defense because it acts as a receptor for microbial ligands during the innate immune response [13]. A functional polymorphism of *CD14* (rs2569190) is associated with an enhanced immune response, and thereby affects the risk of RTIs [14, 15]. This suggests that these genetic variants may affect the occurrence of RTIs and environmental factors may exaggerate the effects of these genetic variations.

Therefore, we hypothesized that there are associations between exposure to perinatal anxiety and genetic variants involved in antioxidant defense and innate immunity, and these interactions influence the RTI risk in offspring, especially during early life. The first aim of this study was to examine the association between perinatal anxiety and RTI occurrence during the first year of life. Second, the effect of the interaction between exposure to perinatal anxiety and genetic variants of some GST subfamily genes and *CD14* (rs2569190) on the occurrence of RTIs was explored.

## Methods

### Participants

Data was used from the COhort for Childhood Origin of Asthma and allergic disease (COCOA), a prospective birth cohort study that aimed to investigate the effects of environmental and genetic factors during early life on the development of allergic diseases and children’s health [16–18]. Women in the third trimester of pregnancy were enrolled from January 2009 to September 2011. The questionnaire used in this study included items on the State-Trait Anxiety Inventory (STAI). Pregnant women with pregnancy-associated complications (gestational diabetes and pregnancy-induced hypertension), high risk for early delivery, and delivery earlier than 36 weeks have been excluded. Neonates were excluded if they needed oxygen therapy, or had congenital anomalies including congenital diaphragmatic hernia, congenital heart disease, congenital lung diseases, or severe systemic diseases. More details on this cohort study are described elsewhere [19].

Among 734 eligible women, 71 refused to participate, 4 were lost to follow up, and 28 were excluded due to preterm delivery. Among a total of 631 (86%), 440 (69.7%) completed the STAI questionnaire. Study participants did not differ from those women not included in the study with regard to key demographic covariates, except for the season of offspring’s birth (Table 1). Information on potential confounders was obtained from a parental-reported questionnaire at the 36th week of pregnancy and medical records at the child’s birth. Questionnaires assessed maternal health problems including allergic diseases (atopic dermatitis, allergic rhinitis, or asthma), socioeconomic status (income and educational levels), and prenatal environmental factors such as tobacco smoking exposure at 36 weeks of gestation. The newborn’s sex and weight, gestational age, season of birth, and health conditions were obtained by a questionnaire after birth.

**Table 1:** **Characteristics of the study population and subjects not included in the current analysis**

	Women included in this study		Women not included in this study		P-value
	***n***	Mean ± SD or ***n***(%)	***n***	Mean ± SD or ***n***(%)	
Covariates (mother)	440		193		
Maternal age (years)		32.5 ± 3.55		32.4 ± 3.45	0.736
BMI (kg/m^2^)		20.7 ± 2.67		20.7 ± 2.60	0.754
Maternal educational level					
Secondary school		31 (7.0%)		21 (10.9%)	0.202
College or University		289 (65.7%)		116 (60.1%)	
Graduate school		120 (27.3%)		56 (29.0%)	
Environmental tobacco exposure		265 (60.8%)		105 (54.4%)	0.171
History of any allergic disorder (AD, AR, Asthma)		124 (28.2%)		59 (30.7%)	0.568
Covariates (father)	410				
History of any allergic disorder (AD, AR, Asthma)		115 (26.1%)		51 (26.4%)	0.939
Covariates (infant)	440		193		
Sex: Girl		217 (49.3%)		85 (44.2%)	0.227
Birth weight (g)		3143.9 ± 422.01		3198.3 ± 395.43	0.153
Season of birth					
Spring		117 (26.6%)		28 (14.5%)	< 0.001
Summer		117 (26.6%)		26 (13.5%)	
Autumn		96 (21.8%)		91 (47.2%)	
Winter		110 (25.0%)		48 (24.9%)	
Mode of delivery: Caesarean section		134 (34.4%)		68 (35.2%)	0.793
Gestational age (months)		39.2 ± 1.08		39.2 ± 1.09	0.968
Presence of siblings		198 (45.0%)		83 (43.0%)	0.705
Daycare attendance during the first year of life		105 (23.9%)		43 (22.3%)	0.740
Exposure to perinatal maternal anxiety	440		N/A		
STAI score		41.0 ± 8.47 (20–71)			
Physician diagnosed respiratory tract infection during the first year of life					
Upper respiratory tract infection		337 (76.6%)			
Lower respiratory tract infection		65 (14.8%)			
Bronchiolitis		48 (10.9%)			

*AD*, atopic dermatitis; *AR*, allergic rhinitis; *N/A*, not applicable; *STAI*, State-Trait Anxiety Inventory.

Written informed consent was obtained from all women and the study was approved by Asan Medical Center (IRB No. 2008-0616), Samsung Medical Center (IRB No. 2009-02-021), Yonsei University (IRB No. 4-2008-0588), and CHA Medical Center (IRB No. 2010-010).

### Anxiety assessment

Data on perinatal maternal anxiety were obtained by self-report at the 36th week of pregnancy. Instead of using STAI, the Korean version of STAI (K-STAI) was used, which consists of two subscales, namely State Anxiety (anxiety about an event) and Trait Anxiety (anxiety levels as a personal characteristic). The Trait Anxiety subscale (STAI-T) was used in this study because it reflects the baseline anxiety levels of the subjects rather than a transient anxiety state. STAI-T is a 20 item questionnaire scored on a four-point Likert-type scale that measures a general tendency to be anxious [20]. Scores range from 20 to 80, with a higher score indicating a more severe anxiety level. K-STAI exhibits excellent psychometric properties [21] and its internal consistency has been reported as having a Cronbach’s α coefficient of 0.91 [22]. In this study, the reliability coefficient (Cronbach’s α) of STAI-T was 0.92.

### Assessment of 1 year outcome variables

The infants were examined at 1 year of age with at least one parent present at follow-up visits. RTIs were assessed by parent-reported doctor diagnosis during the first year of life. RTIs were further categorized as upper (URTIs) and lower (LRTIs) RTIs. URTIs included common colds, sinusitis, otitis media, and croup; LRTIs included pneumonia, tracheobronchitis, and bronchiolitis.

### DNA isolation and genotyping

Genomic DNA was extracted from the buffy coat of the cord blood at delivery using the Gentra® Puregene® Blood kit (Qiagen, Maryland, USA), as recommended by the manufacturer. Genotyping of *CD14* (rs2569190) and *GSTP1* (rs1695) was conducted using a TaqMan assay (ABI, Foster City, CA, USA). Assay identification numbers for *CD14* and *GSTP1* were C_16043997_10 and C_3237198_20, respectively. Copy number variations (CNV) of *GSTM1* and *GSTT1* were measured by real-time polymerase chain reaction (RT-PCR). The primers and probe for *GSTM1* and *GSTT1* were synthesized according to Brasch-Andersen C et al. [23]. Reactions were performed as a triplex, with RNAse P as the reference gene. The allele frequencies of the polymorphisms in *GSTP1* (rs1695), *GSTM1*, *GSTT1*, and *CD14* (rs2569190) were in Hardy-Weinberg equilibrium (*P* > 0.1).

### Statistical analysis

The association between perinatal maternal anxiety levels and RTIs (URTIs, LRTIs, and bronchiolitis) during the first year of life was examined using logistic regression. Adjustments were made for baseline demographic factors (child’s sex and maternal age) as well as known risk factors for respiratory infections, including maternal educational level, exposure to tobacco smoking during the first year, and presence of maternal and paternal allergic diseases [24, 25].

The standard deviation (SD) for the STAI score in the regression analyses was 8.47. Parameter estimates were multiplied by the SD of the STAI score, and for 95% confidence interval (CI), the following equation was used: BETA*(SD of STAI score) ± 1.96*SE*(SD of STAI score). In logistic regression analysis, the estimates were exponentiated to obtain the estimate and 95% CI of the odds ratio (OR).

The combined effects of perinatal anxiety and genetic polymorphisms on RTI risk were investigated by multivariate logistic regression analysis. To analyze the combined effect of the two factors, STAI scores were divided into two categories on the basis of mean levels and genetic polymorphisms were classified according to *GSTT1* null or present, *GSTM1* null or present, and polymorphisms of *GSTP1* (rs1695) and *CD14* (rs2569190). All statistical analyses were performed using SAS for Windows (version 9.2).

## Results

### Demographics

Table 1 summarizes the descriptive statistics across the total sample. There were no significant differences in maternal age, maternal education level, infants’ sex, mode of delivery, presence of infants’ siblings, and daycare attendance of infants between pregnant women included in this study and those excluded. Overall, the mean maternal age was 32.5 years. Exposure to tobacco smoking during pregnancy was reported by 60.8% of the women. In mothers, 28.2% had a history of allergic disease, whereas in spouses the proportion was 26.1%. The STAI score demonstrated a normal distribution with mean ± SD of 41.0 ± 8.47. Approximately half (49.3%) of the offspring were girls, with a mean gestational age of 39.2 weeks. Births were evenly distributed between the four seasons (26.6% in spring, 26.6% in summer, 21.8% in autumn, and 25.0% in winter). Approximately one third of births (34.4%) were delivered by caesarean section.

### Association between perinatal maternal anxiety and development of RTIs during the first year of life

**Figure 1:**
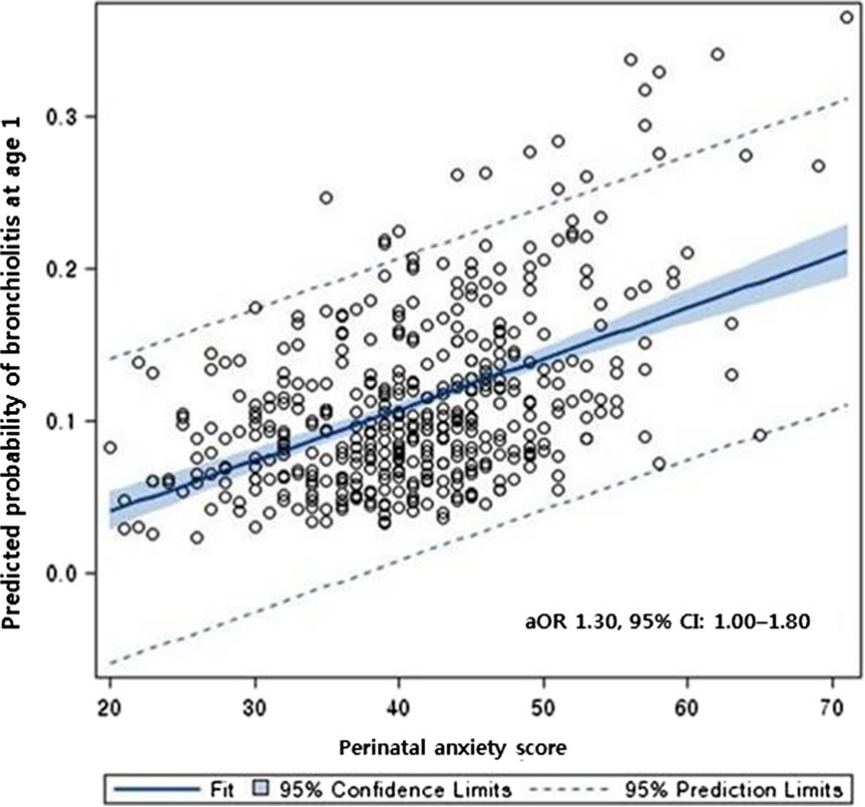
**Relationship between perinatal maternal anxiety levels and predicted probability of bronchiolitis at 1 year of age.**

High levels of perinatal maternal anxiety were associated with an increased risk of LRTIs, especially bronchiolitis, but not with total URTIs (Table 2). For a 1 SD increase in STAI score, the odds of LRTI risk during the first year of life increased by 1.35 times (95% CI: 1.01–1.78). The crude association between perinatal anxiety levels and the development of bronchiolitis was weakly significant (OR 1.35, 95% CI: 1.00–1.81; adjusted OR [aOR] 1.30, 95% CI: 1.00–1.80). However, perinatal anxiety did not increase the risk of URTIs (aOR 1.10, 95% CI: 0.87–1.39). In addition, high levels of perinatal maternal anxiety were not significantly associated with the occurrence of other subtypes of RTIs except bronchiolitis. Figure 1 represents the predicted probability of bronchiolitis at 1 year of age for each observed value of perinatal anxiety score in the multivariate model.

**Table 2:** **Odds ratio and 95% confidence interval for perinatal anxiety predicting RTIs at age 1 year**

Exposure	Outcome	Unadjusted		Adjusted ^*^	
		OR ^†^	95% CI	OR	95% CI
STAI (SD)	URTI	1.04	(0.84–1.30)	1.10	(0.87–1.39)
	LRTI	1.31^‡^	(1.01–1.71)	1.35^‡^	(1.01–1.78)
	Bronchiolitis	1.35^‡^	(1.00–1.81)	1.30^§^	(1.00–1.80)

*CI*, confidence interval; *OR*, odds ratio; *RTI*, respiratory tract infection; *SD*, standard deviation; *STAI*, State-Trait Anxiety Inventory.

^*^Adjusted for child’s sex, season of birth, maternal age, education level, exposure to tobacco smoking during the first year of life, history of maternal allergic diseases and history of any paternal allergic diseases (atopic dermatitis, allergic rhinitis, or asthma).

^†^Logistic regression models were used to calculate OR and 95% CI.

^‡^ *p*-value < 0.05.

^§^ *p*-value < 0.10.

### Combined effect between perinatal maternal anxiety levels and *GSTP1*(rs1695), *GSTT1*, *GSTM1*polymorphisms on the development of RTIs during the first year of life

Genetic polymorphisms in *GSTP1* (rs1695), *GSTT1* or *GSTM1* did not increase the risk of any types of RTIs (data not shown). Exposure to high levels of perinatal anxiety was associated with an increased risk for LRTIs, especially bronchiolitis, in infants with the AG + GG genotype of *GSTP1* (rs1695), compared to infants with exposure to low levels of perinatal anxiety and with the AA genotype (aOR 2.61, 95% CI: 1.09–6.22; aOR 3.36, 95% CI: 1.25–9.03, respectively) (Table 3). Analysis of the three genotypes showed that, infants with the GG genotype of *GSTP1* (rs1695) had a significantly increased risk of LRTIs, especially bronchiolitis, during the first year of life (aOR 22.04, 95% CI: 4.79–101.47; aOR 14.61, 95% CI: 3.23–66.11, respectively) (Additional file 1: Figure S1).

**Table 3:** **Interactions between maternal perinatal anxiety (STAI score) and** ***GSTP1*** **(rs1695) polymorphism on the development of respiratory tract infection during the first year of life**

		URTI ^*^			LRTI ^†^			Bronchiolitis		
STAI	***GSTP1***	URTI diagnosis (-)	URTI diagnosis (+)	aOR ^‡^ (95% CI)	LRTI diagnosis (-)	LRTI diagnosis (+)	aOR ^‡^ (95% CI)	Bronchiolitis diagnosis (-)	Bronchiolitis diagnosis (+)	aOR ^‡^ 95% CI
low (≤41)	AA	36	111	1	133	14	1	138	9	1
low (≤41)	AG + GG	17	43	1.10 (0.53–2.27)	51	9	1.66 (0.64–4.26)	52	8	2.14 (0.74–6.15)
high (>41)	AA	31	90	1.25 (0.68–2.30)	101	20	1.99 (0.92–4.29)	106	15	2.07 (0.83–5.16)
high (>41)	AG + GG	13	53	1.91 (0.86–4.25)	52	14	2.61 (1.09–6.22)	54	12	3.36 (1.25–9.03)

*aOR*, adjusted odds ratio; *CI*, confidence interval; *GSTP1,* glutathione *S*-transferase P1; *LRTI,* lower respiratory tract infection; *RTI*, respiratory tract infection; *URTI,* upper respiratory tract infection; *STAI*, State-Trait Anxiety Inventory.

^*^URTI includes common colds, sinusitis, otitis media, and croup.

^†^LRTI includes pneumonia, tracheobronchitis, and bronchiolitis.

^‡^Adjusted for infant’s sex, season of birth, maternal age, education level, prenatal exposure to tobacco smoking, history of any maternal allergic diseases and history of any paternal allergic diseases (atopic dermatitis, allergic rhinitis, or asthma).

In addition, infants with both the *GSTT1*-null genotype and exposure to high levels of perinatal anxiety were associated with an increased risk for LRTIs, especially bronchiolitis, compared to infants with both the *GSTT1*-present genotype and exposure to low levels of perinatal anxiety (aOR 2.47, 95% CI: 1.04–5.89; aOR 2.79, 95% CI: 1.05–7.43) (Table 4). In terms of *GSTM1*, exposure to high levels of perinatal anxiety increased the risk of LRTIs during the first year of life, regardless of the genotypes (present genotype: aOR 2.95, 95% CI: 1.06–8.17; null genotype: aOR 2.60, 95% CI: 1.01–6.66) (Additional file 2: Table S1).

**Table 4:** **Interactions between maternal perinatal anxiety (STAI score) and copy number variation of** ***GSTT1*** **on the development of respiratory tract infection during the first year of life**

		URTI ^*^			LRTI ^†^			Bronchiolitis		
STAI	***GSTT1***	URTI diagnosis (-), n	URTI diagnosis (+), n	aOR ^‡^ (95% CI)	LRTI diagnosis (-), n	LRTI diagnosis (+), n	aOR ^‡^ (95% CI)	Bronchiolitis diagnosis (-), n	Bronchiolitis diagnosis (+), n	aOR ^‡^ (95% CI)
low (≤41)	CNV ≥ 1	16	76	1	82	10	1	85	7	1
low (≤41)	CNV = 0	37	76	0.43 (0.21–0.88)	102	11	0.86 (0.33–2.21)	104	9	0.97 (0.33–2.83)
high (>41)	CNV ≥ 1	22	65	0.66 (0.30–1.45)	75	12	1.29 (0.50–3.33)	79	8	1.13 (0.37–3.34)
high (>41)	CNV = 0	22	77	1.02 (0.46–2.26)	77	22	2.47 (1.04–5.89)	80	19	2.79 (1.05–7.43)

*aOR*, adjusted odds ratio; *CI*, confidence interval; *CNV*, copy number variation; *GSTT1,* glutathione *S*-transferase T1; *LRTI,* lower respiratory tract infection; *RTI*, respiratory tract infection; *URTI,* upper respiratory tract infection; *STAI*, State-Trait Anxiety Inventory.

^*^URTI includes common colds, sinusitis, otitis media, and croup.

^†^LRTI includes pneumonia, tracheobronchitis, and bronchiolitis.

^‡^Adjusted for infant’s sex, season of birth, maternal age, education level, prenatal exposure to tobacco smoking, history of any maternal allergic diseases and history of any paternal allergic diseases (atopic dermatitis, allergic rhinitis, or asthma).

The subject number of this analysis is different from those in Table 3 due to the failure of simultaneous genotyping of *GSTP1* (rs1695) and *GSTT1* in several subjects.

### Combined effect between exposure to perinatal maternal anxiety and *CD14*(rs2569190) polymorphism on the development of RTIs during the first year of life

Infants with the TT genotype of *CD14* (rs2569190) had an increased risk of URTIs and LRTIs, especially bronchiolitis, if they were exposed to high levels of perinatal anxiety, compared with infants exposed to low levels of perinatal anxiety (aOR 2.51, 95% CI: 1.01–6.24; aOR 4.60, 95% CI: 1.29–16.41; aOR 4.31, 95% CI: 1.17–15.79, respectively) (Additional file 3: Table S2).

## Discussion

### Main findings

In this study, a positive association was found between exposure to high levels of perinatal anxiety and development of RTIs in offspring during the first year of life. The risk of LRTIs, especially bronchiolitis, during early infancy was influenced by additive effects between exposure to perinatal anxiety and polymorphisms in antioxidant-related genes. To the best of our knowledge, this is the first study examining the gene-environment interactions with respect to perinatal anxiety on the development of RTIs during early life.

Exposure to high levels of perinatal anxiety affected the development of RTIs, especially bronchiolitis, differently during infancy, depending on the polymorphisms of ROS detoxification genes such as *GSTP1* (rs1695) and *GSTT1*. The combined effect of exposure to perinatal maternal anxiety and ROS detoxification gene polymorphisms on the occurrence of LRTIs, especially bronchiolitis, may be partially attributable to the increased ROS generation caused by high levels of perinatal maternal anxiety. Perinatal anxiety increases glucocorticoid levels in both mother and fetus, and this induces superoxide production [26]. The capacity to effectively detoxify ROS is affected by polymorphisms of ROS detoxification genes [27, 28]. The increased risk of LRTIs, especially bronchiolitis, in infants with the AG + GG genotype of *GSTP1* (rs1695) or the *GSTT1*-null genotype after exposure to high levels of perinatal anxiety may be explained by the reduced enzymatic activity in the AG + GG genotype of *GSTP1* (rs1695) and by the loss of enzymatic activity in the *GSTT1*-null genotype [27, 28]. Since GST subfamily genes are expressed mainly in the respiratory tract and oxidative stress is increased during LRTIs, especially bronchiolitis [29], genetic variants of the GST subfamily may influence the risk of LRTIs, especially bronchiolitis.

### Possible underlying mechanisms

Psychological stress and anxiety may affect the balance of oxidant and antioxidant states and thereby influence an individual’s health [30, 31]. In response to stress, serum glucocorticoid levels rise to modulate the stress response [32]. Glucocorticoid levels affect antioxidant enzyme levels, and antioxidant enzyme activity in the lungs begins in late gestation [33, 34]. Considering the impact of ROS in fetal programming and variants of ROS-related genes on the capacity to detoxify ROS [11, 12], the increased susceptibility to RTIs in offspring exposed to perinatal anxiety might be explained by their decreased ability to mitigate the oxidative stress generated during RTIs [35].

An experimental study showed that prenatal maternal stress influences epigenetic re-programming by altering the expression of microRNAs involved in the stress response, oxidative stress, and metabolism [6]. In addition, prenatal anxiety-related immune and oxidative responses cause telomere erosion, possibly increasing the risk of infection [36, 37]. On the basis of these findings, we suggest that perinatal anxiety influences the susceptibility to RTIs in offspring during critical periods in combination with genetic variants of ROS-related genes or through epigenetic mechanisms. Additional studies are needed to clarify the underlying mechanisms.

Previous studies showed that the T allele of *CD14-*159C/T is associated with an enhanced transcriptional activity and increased soluble CD14 levels, which are markers of monocyte and macrophage activation [38, 39]. The positive relationship between perinatal anxiety level and risk of RTIs in infants with the TT genotype of *CD14* (rs2569190) might be due to the essential role of CD14 as an initiator of innate immunity against viral infections in the respiratory tract [14]. Alterations in cellular and humoral immune responses caused by the interactions between exposure to high levels of perinatal maternal stress and genetic variants of *CD14* might partially explain these findings [40, 41].

In this study, it was proposed that perinatal maternal anxiety differentially affects the risk of RTIs depending on their anatomic location in the respiratory tract. Increased glucocorticoid levels during the fetal period may affect the development and function of the lungs during early life [42]. Differences in defense mechanisms, and the more severe characteristics of LRTIs than URTIs, may account for the increased risk of LRTIs [43, 44]. In addition, functional differences attributable to the genetic variants in ROS-related genes may add an increased susceptibility to LRTIs, especially bronchiolitis, in affected infants [27, 41].

Hospitalization expenditures because of bronchiolitis during infancy has increased [45]. A history of bronchiolitis in infancy is associated with adverse respiratory outcome such as reduced lung function and development of asthma in later life [46, 47]. With the prevention of bronchiolitis during early life, health care costs and adverse respiratory health can be avoided.

### Potential limitations

The current study has a few limitations. First, the sample size in this study is relatively small because this study is an ongoing prospective birth cohort study. Although the statistical power was weak to analyze the associations with respect to each type of LRTI except bronchiolitis due to the small sample size, this study indicates the importance of the effect of perinatal maternal anxiety on the occurrence of RTIs during early life. Further large-scale studies are needed to validate the association between perinatal anxiety and LRTIs during infancy, especially bronchiolitis, which is associated with adverse respiratory outcomes such as asthma in later life. Second, mechanisms underlying the additive effects between perinatal anxiety and variants of antioxidant defense genes on the occurrence of LRTIs, especially bronchiolitis, were not explored. However, previously reported findings were used to try and explain the underlying pathophysiology. Also, only one single nucleotide polymorphism (SNP) was analyzed for each gene; however, this selection was based on previous studies demonstrating an association between these SNPs and the development of respiratory illnesses [29, 34, 38]. Finally, approximately 30% of the participants did not report their anxiety status and were thus excluded from the analysis.

## Conclusion

This study suggests that exposure to perinatal maternal anxiety is a significant risk factor for the development of bronchiolitis in the first year of life. It indicates that variants in antioxidant defense genes modulate the effect of *in utero* exposure to perinatal anxiety on infant susceptibility to bronchiolitis during critical periods. In view of the fact that bronchiolitis during early infancy is an economic and medical burden and is associated with an increased risk of recurrent wheezing and asthma in later life, efforts to decrease perinatal anxiety may help prevent bronchiolitis during infancy, especially in genetically susceptible infants.

## Authors’ information

Eun Lee and Hyoung Yoon Chang as co-first authors.

## Electronic supplementary material

Additional file 1: Figure S1: Effect of *GSTP1* (rs1695) polymorphisms on respiratory tract infections (RTIs) according to perinatal maternal anxiety levels. (A) The risk of upper RTIs (URTIs) during the first year of life. (B) The risk of lower RTIs (LRTIs) during the first year of life. (C) The risk of bronchiolitis during the first year of life. (DOC 3 MB)

Additional file 2: Table S1: Interactions between maternal perinatal stress (STAI score) and copy number variations of *GSTM1* on the development of respiratory tract infection during the first year of life. (DOC 32 KB)

Additional file 3: Table S2: Interactions between maternal perinatal stress (STAI score) and *CD14* (rs2569190) polymorphism on the development of respiratory tract infection during the first year of life. (DOC 32 KB)

## Abbreviations

CI: Confidence interval
COCOA: COhort for childhood origin of asthma and allergic disease
GSTM1: Glutathione *S*-transferase M1
GSTP1: Glutathione *S*-transferase P1
GSTT1: Glutathione *S*-transferase T1
LRTIs: Lower respiratory tract infections
PCR: Polymerase chain reaction
ROS: Reactive oxygen species
RSV: Respiratory syncytial virus
RTI: Respiratory tract infection
SD: Standard deviation
STAI: State-Trait Anxiety Inventory
URTIs: Upper respiratory tract infections.
