# Supplementary material for: The effect of perinatal anxiety on bronchiolitis is influenced by polymorphisms in ROS-related genes
Source: BMC Pulm Med. 2014 Sep 29;14:154. doi: 10.1186/1471-2466-14-154 (PMC4196140; doi:10.1186/1471-2466-14-154)
Supplement: Supplementary file 1 — Additional file 1: Figure S1: Effect of GSTP1 (rs1695) polymorphisms on respiratory tract infections (RTIs) according to perinatal maternal anxiety levels. (A) The risk of upper RTIs (URTIs) during the first year of life. (B) The risk of lower RTIs (LRTIs) during the first year of life. (C) The risk of bronchiolitis during the first year of life. (DOC 3 MB) [file 12890_2014_593_MOESM1_ESM.doc]

**Additional file 1: Figure S1.** Effect of *GSTP1* (rs1695) polymorphisms on respiratory tract infections (RTIs) according to perinatal maternal anxiety levels. (A) The risk of upper RTIs (URTIs) during the first year of life. (B) The risk of lower RTIs (LRTIs) during the first year of life. (C) The risk of bronchiolitis during the first year of life.


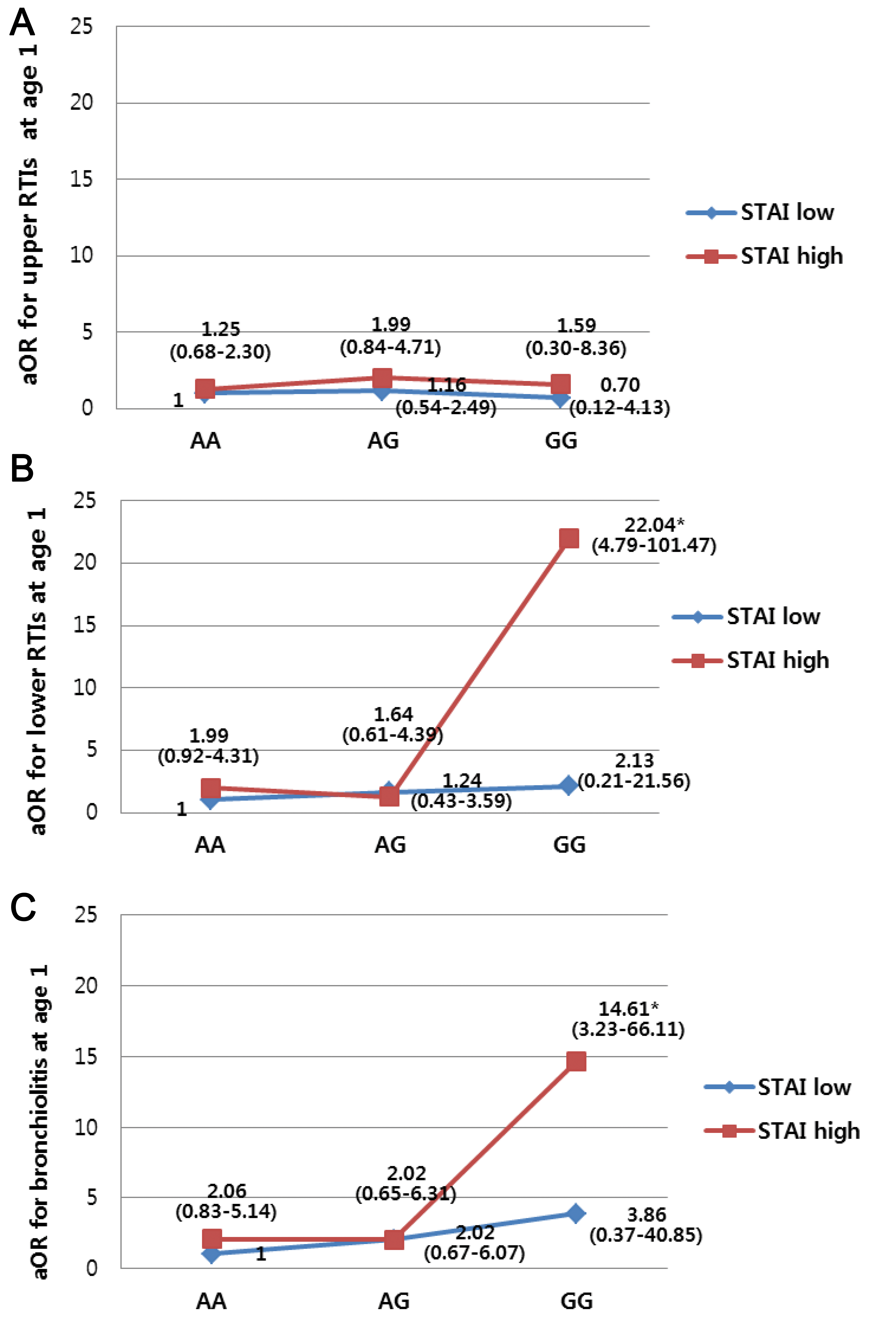


STAI, State-Trait Anxiety Inventory.
