# Supplementary material for: The effect of perinatal anxiety on bronchiolitis is influenced by polymorphisms in ROS-related genes
Source: BMC Pulm Med. 2014 Sep 29;14:154. doi: 10.1186/1471-2466-14-154 (PMC4196140; doi:10.1186/1471-2466-14-154)
Supplement: Supplementary file 3 — Additional file 3: Table S2: Interactions between maternal perinatal stress (STAI score) and CD14 (rs2569190) polymorphism on the development of respiratory tract infection during the first year of life. (DOC 32 KB) [file 12890_2014_593_MOESM3_ESM.doc]

***Additional file 3: Table S2*** *Interactions between maternal perinatal stress (STAI score) and CD14 (rs2569190) polymorphism on the development of respiratory tract infection during the first year of life*

|  | | URTI^*^ | | | LRTI^†^ | | | Bronchiolitis | | |
| --- | --- | --- | --- | --- | --- | --- | --- | --- | --- | --- |
| STAI | *CD14* | URTI diagnosis (-), n | URTI  Diagnosis (+), n | aOR^‡^  (95% CI) | LRTI diagnosis  (-), n | LRTI diagnosis (+), n | aOR^‡^  (95% CI) | Bronchiolitis diagnosis (-), n | Bronchiolitis diagnosis (+), n | aOR^‡^  (95% CI) |
| low (≤ 41) | TT | 21 | 43 | 1 | 60 | 4 | 1 | 60 | 4 | 1 |
| low (≤ 41) | TC+CC | 31 | 110 | 1.89  (0.93–3.83) | 123 | 18 | 2.22  (0.69–7.14) | 128 | 13 | 1.58  (0.47–5.31) |
| high (> 41) | TT | 13 | 44 | 2.51  (1.01–6.24) | 46 | 11 | 4.60  (1.29–16.41) | 47 | 10 | 4.31  (1.17–15.79) |
| high (> 41) | TC+CC | 31 | 96 | 1.90  (0.91–3.94) | 105 | 22 | 3.01  (0.94–9.70) | 110 | 17 | 2.11  (0.63–7.04) |

*aOR*, adjusted odds ratio; *CI*, confidence interval; *LRTI,* lower respiratory tract infection; *RTI*, respiratory tract infection; *URTI,* upper respiratory tract infection; *STAI*, State-Trait Anxiety Inventory.

^*^URTI includes common colds, sinusitis, otitis media, and croup.

^†^LRTI includes pneumonia, tracheobronchitis, and bronchiolitis.

^‡^Adjusted for child’s sex, season of birth, maternal age, education level, prenatal exposure to tobacco smoking, history of any maternal allergic diseases and history of any paternal allergic diseases (atopic dermatitis, allergic rhinitis, or asthma).
